# Polynomial probability distribution estimation using the method of moments

**DOI:** 10.1371/journal.pone.0174573

**Published:** 2017-04-10

**Authors:** Joakim Munkhammar, Lars Mattsson, Jesper Rydén

**Affiliations:** 1 BEESG, Department of Engineering Sciences/Uppsala University, Uppsala, Sweden; 2 Nordita, KTH Royal Institute of Technology and Stockholm University, Stockholm, Sweden; 3 Department of Mathematics, Uppsala University, Uppsala, Sweden; Universita degli Studi del Piemonte Orientale Amedeo Avogadro, ITALY

## Abstract

We suggest a procedure for estimating *N*th degree polynomial approximations to unknown (or known) probability density functions (PDFs) based on *N* statistical moments from each distribution. The procedure is based on the method of moments and is setup algorithmically to aid applicability and to ensure rigor in use. In order to show applicability, polynomial PDF approximations are obtained for the distribution families Normal, Log-Normal, Weibull as well as for a bimodal Weibull distribution and a data set of anonymized household electricity use. The results are compared with results for traditional PDF series expansion methods of Gram–Charlier type. It is concluded that this procedure is a comparatively simple procedure that could be used when traditional distribution families are not applicable or when polynomial expansions of probability distributions might be considered useful approximations. In particular this approach is practical for calculating convolutions of distributions, since such operations become integrals of polynomial expressions. Finally, in order to show an advanced applicability of the method, it is shown to be useful for approximating solutions to the Smoluchowski equation.

## Introduction

Estimating PDFs is essential in applied statistical analysis in many diverse fields of science, for instance for a few examples from our own experience in engineering [1, 2], physics [3, 4] and the Earth sciences [5, 6]. With knowledge of the PDF, further statistical problems can be tackled, such as for instance estimation of quantiles, and it can be used to construct stochastic models for various applications [1, 2].

In applied statistics, a distribution family is usually decided upon for a particular situation, and fitting a PDF then means determination of parameters by some estimation technique. Several common parametric methods such as the method of maximum likelihood and the method of moments are then well-known examples [7]. In case there is no prior knowledge of the distribution family, other methods can be used, for instance kernel-density estimation (see e.g. [8]) or B-splines (see e.g [9], chapter 6). It is also possible to obtain approximation by means of series expansions, e.g. Gram–Charlier series and Edgeworth series [10, 11]. However, such expansions are often considered useful only in cases of moderate skewness [11]. Generally, the complexity of series approximation methods and the simplicity of traditional distribution fitting has inspired the use of series approximation methods in applied statistics, albeit to a limited level of use. See p.107-117 [12] for a review of approximation methods in statistics and [13] for a recent review of series approximation methods in statistics.

The present paper suggests an algorithm for estimating an *N*-order polynomial approximation of a PDF, based only on *N* moments of the PDF. Polynomial approximations for example distributions are given and a comparison with existing PDF series expansions is made. In addition to this, regarding more advanced problems, an example is used to illustrate that the procedure can be useful for approximating solutions to the Smoluchowski equation for such cases where moments of the solution are available.

## Procedure

The method of moments estimates parameters of a predefined distribution *f* by equating moments of sample values and moments of the distribution, see p.467 [14]. This forms a system of equations which is often not analytically solvable, see p.467 [14], p.816 [15].

The procedure below estimates coefficients for a polynomial probability distribution approximation of the PDF *f*. This is done, via the method of moments, by equating the known moments of *f* with moments of the polynomial approximation of *f*.

The procedure is defined by the following algorithm:

1. Define a goodness-of-fit test T (for example a Kolmogorov-Smirnov test) for the PDF approximation.
2. Choose a real interval [*a*′, *b*′] on which the approximation should hold good.
3. Choose a polynomial degree *N* ∈ [0, 1, 2, 3, …] for approximating a not necessarily known PDF *f*.
4. Ensure that *n*th order statistical moments for *n* ∈ [0, …, *N*] of *f* based on a real valued interval [*a*, *b*] are available. ([*a*, *b*] may equal [*a*′, *b*′], but not necessarily)
5. As an approximation to *f*, define an *N*th order polynomial *P* on the real interval *x* ∈ [*a*′, *b*′]:
  $$P\left(N,x\right)\equiv w_{0}+w_{1}x+w_{2}x^{2}+w_{3}x^{3}+\cdots +w_{N}x^{N}$$ (1)
  where *w*_*n*_, for each *n* ∈ [0, …, *N*], are unknown weights.
6. When solvable, the equation system resulting from equating the known *i*th moment of *f* on the interval [*a*, *b*] with the unknown *i*th moment of the approximation *P*, for *i* ∈ [0, …, *N*] and interval [*a*, *b*], is used to determine *w*_*n*_ for *n* ∈ [0, …, *N*]. If not solvable, repeat from step 2 or 3 with increased (or decreased) *N* or changed [*a*, *b*].
7. To qualify as a PDF, *P*(*N*, *x*), defined on [*a*′, *b*′], must be non-negative on [*a*′, *b*′]. If this is not met repeat from step 3 with increased (or decreased) *N* or changed [*a*, *b*].
8. Also, to satisfy the conditions for a PDF on [*a*′, *b*′], *P*(*N*, *x*) should be normalized by $P\left(N,x\right)\to P\left(N,x\right)/\int_{a^{\prime }}^{b^{\prime }}P\left(N,x\right)\;dx$ so that $\int_{a^{\prime }}^{b^{\prime }}P\left(N,x\right)\;dx=1$.
9. The procedure holds good if the polynomial approximation *P*(*N*, *x*), for the predefined measure of goodness-of-fit T, sufficiently approximates *f* on [*a*′, *b*′]. For improved fit, repeat the procedure from step 3 with increased (or decreased) *N* or different choices of [*a*, *b*].

In step 1 it is instructive to adopt a goodness-of-fit test such as for example a Kolmogorov-Smirnov test or Akaike’s information criterion in order to ensure goodness-of-fit and applicability compared with other distributions, and set proper limits for pass and fail depending on application, see e.g. [16] and [17] for more information on that.

Given a defined goodness-of-fit test and a real interval [*a*′, *b*′] for the distribution steps 3 and 4 certify the existence of *N* statistical moments. Consider a random variable *X* with probability density function *f*(*x*), then the statistical moment of order *k* is defined by:

$$E_{f}\left[X^{k}\right]\equiv \int_{a}^{b}x^{k}f\left(x\right)dx,$$ (2)

where *a*, *b* are real valued limits for the statistical moment. With only a data set {*x*_1_, *x*_2_, *x*_3_, …, *x*_*M*_} available, the sample moment of order *k* is given by:

$$E_{f}\left[X^{k}\right]\equiv \frac{1}{M}\sum_{i=1}^{M}x_{i}^{k}.$$ (3)

Although the distribution *f* is considered continuous, it is not a requirement of the procedure. However, step 5 in the algorithm is motivated by the use of a continuous distribution since the Weierstrass approximation theorem shows that a continuous function on an interval [*a*, *b*] can be approximated arbitrarily good with a polynomial [18, 19]. With step 5 and Eq (2) it is possible to obtain the statistical moment of order *n* for the approximate polynomial *P*(*N*, *x*):

$$\begin{array}{r} E_{P}\left[X^{k}\right]=\int_{a}^{b}x^{k}P\left(N,x\right)\text{d}x=\\ =\left(w_{0}\frac{x^{k+1}}{k+1}+w_{1}\frac{x^{k+2}}{k+2}+\cdots +w_{N}\frac{x^{k+N+1}}{k+N+1}\right){|}_{a}^{b}, \end{array}$$ (4)

Via step 6 the moments *E*_*f*_[*X*^*n*^] of *f* are equated with the moments *E*_*P*_[*X*^*n*^] of the approximation *P*. With the polynomial approximation this can be setup in a linear equation system in *w*_*n*_:

Mw=E, (5)

where:

$$M=\left[\begin{array}{cccc} b-a & \frac{b^{2}-a^{2}}{2} & \cdots & \frac{b^{N+1}-a^{N+1}}{N+1}\\ \frac{b^{2}-a^{2}}{2} & \frac{b^{3}-a^{3}}{3} & \cdots & \frac{b^{N+2}-a^{N+2}}{N+2}\\ \frac{b^{3}-a^{3}}{3} & \frac{b^{4}-a^{4}}{4} & \cdots & \frac{b^{N+3}-a^{N+3}}{N+3}\\ \vdots & \vdots & \ddots & \vdots \\ \frac{b^{N}-a^{N}}{N} & \frac{b^{N+1}-a^{N+1}}{N+1} & \cdots & \frac{b^{N+N}-a^{N+N}}{2N} \end{array}\right],\;w=\left[\begin{array}{c} w_{0}\\ w_{1}\\ w_{2}\\ \vdots \\ w_{N} \end{array}\right],\;E=\left[\begin{array}{c} E_{f}\left[X^{0}\right]\\ E_{f}\left[X^{1}\right]\\ E_{f}\left[X^{2}\right]\\ \vdots \\ E_{f}\left[X^{N}\right] \end{array}\right].$$ (6)

By inspection **M** is symmetric and a Hankel matrix [20]. The unknown weights *w*_*n*_ for *n* ∈ [0, …, *N*] constituting the vector **w** can now be obtained by computing the inverse of the matrix **M**:

$$w=M^{-1}E.$$ (7)

It should be noted that since the matrix **M** is a Hankel matrix there exists particular algorithms for computing the inverse of the matrix as long as it is finite [21]. Also, the special case of using *a* = 0 and *b* = 1 makes **M** a Hilbert matrix, for which there exists an explicit expression for inversion, see [22]. Inserting **w** from Eq (7) in the polynomial approximation *P*(*N*, *x*) in Eq (1) brings the polynomial approximation for *f*:

$$P\left(N,x\right)=w\cdot \left[1\;x\;x^{2}\;x^{3}\ldots \;\;x^{N}\right].$$ (8)

The fact that *P*(*N*, *x*) is supposed to approximate a PDF (Step 7) requires that *P*(*N*, *x*) is non-negative on the interval [*a*′, *b*′], which is not generally certified. As described in more detail in step 9 increased (or decreased) *N* or changed [*a*, *b*] could solve this issue. When step 7 is checked, step 8 normalizes the PDF on [*a*′, *b*′], and if *P*(*N*, *x*) satisfies the goodness-of-fit test T (step 9), the procedure is finished.

Step 9 is important since there is no explicit check for convergence inside the procedure, a property not uncommon for series approximations in statistics, see p.114-115 [12]. Also, it is not unlikely that the set of moments might not uniquely describe a distribution, see [23].

With the computed weights *w*_*n*_ the CDF and the statistical moments of the polynomial distribution *P*(*N*, *x*) is in turn possible to calculate analytically using Eq (4).

From a statistical point of view, an interesting property of a polynomial distribution approach is the convolution of distributions. The convolution of PDFs *g*(*x*) and *h*(*x*), both valid for some domain *x* ∈ [*c*, *d*], is defined as:

$$\left(g*h\right)\left(x\right)=\int_{c}^{d}g\left(t\right)h\left(x-t\right)\;dt.$$ (9)

Convolution of probability distributions is a complicated mathematical procedure for most applications. Also, only few distributions can be convoluted analytically and often this is instead handled numerically or approximated with e.g. the central limit theorem, such as in [2]. With the use of polynomial distributions *P*(*N*, *x*) ≡ *w*_0_ + *w*_1_*x* + *w*_2_*x*^2^ + ⋯ + *w*_*N*_*x*^*N*^ and *Q*(*M*, *x*) ≡ *z*_0_ + *z*_1_*x* + *z*_2_*x*^2^ + ⋯ + *z*_*M*_*x*^*M*^ the convolution Eq (9) in turn becomes a polynomial expression:

$$\left(P*Q\right)\left(x\right)=\left(v_{0}+v_{1}x+v_{2}x^{2}+\cdots +v_{N+M}x^{N+M}\right){|}_{a^{\prime }}^{b^{\prime }}$$ (10)

for some computable weights *v*_0_, *v*_1_, …, *v*_*N*+*M*_.

For data on the sum of correlated stochastic variables *X*_1_, *X*_2_, … it is possible to obtain a polynomial approximation of the resultant distribution by using moments from the sum of the stochastic variables. For such situations, which might be multimodal, and generally for more complex distributions, the polynomial estimation procedure simplifies the process which otherwise involves using complicated methods for determining parameters of mixture distributions. See [24] for information on the use of mixture distributions, and [1] for an applied example of mixture distributions in solar engineering.

## Polynomial expansions of known probability distributions

**Fig 1:**
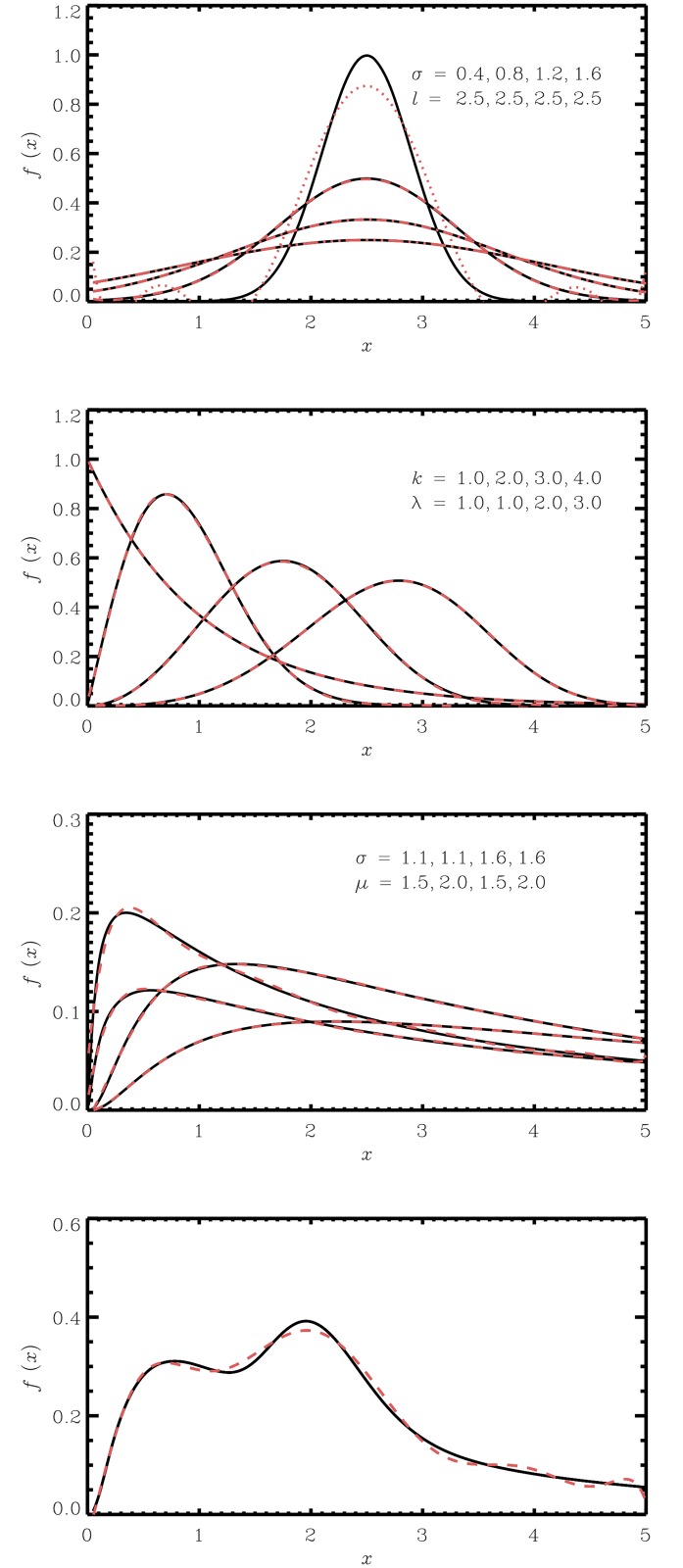
A set of distributions (black line) and polynomial approximations (dashed line) to level 10 for the common distributions: Normal, Weibull, Log-Normal, Bimodal. The polynomial distributions were computed from numerically integrated moments on the interval *a* = 0, *b* = 5.

In Fig 1, we present examples of the procedure applied to commonly used PDFs. For each of three distribution families (Normal, Weibull, Log-Normal), four parameter settings were considered. The moments were directly calculated from explicit expressions for each distribution. Moreover, order *N* = 10 of the polynomial was chosen to ensure a high level goodness-of-fit, however in principle any *N* terms can be used, with varying degree of goodness-of-fit as a result. By visual inspection of the plots, the agreement between the theoretical curve and the polynomial approximation seems satisfactory. Moreover, in Fig 1, we present an example with a bimodal distribution constructed from two Weibull distributions. Also here, the polynomial approximation adequately reproduces the distribution. Note that [*a*′, *b*′] is not explicitly defined for the results presented in Fig 1, but could be used for at least the entire interval displayed in the plots (which equals [*a*, *b*]), in all cases except for the normal distribution with mean 2.5 and variance 0.4 which is negative for some parts of the interval. In that case changed [*a*, *b*] or *N*, or if changed [*a*′, *b*′] would be admissible (such as e.g. *a*′ = 2 and *b*′ = 3), could aid this issue.

**Fig 2:**
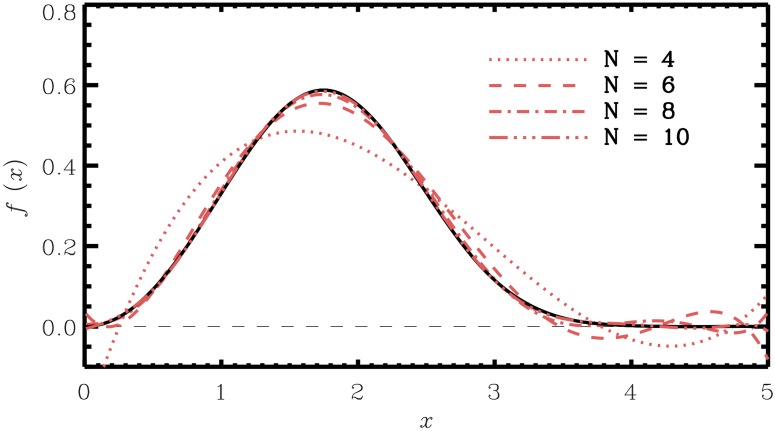
A Weibull distribution and polynomial distributions for certain number of terms *N*. The polynomial distributions were computed from numerically calculated moments on the interval *a* = 0, *b* = 5. In the polynomial distributions, note the wiggle of and negative values when the PDF is close to zero.

The convergence rate with increasing order of polynomials is illustrated with an example distribution in Fig 2. In this example a two-parameter Weibull distribution with shape parameter *k* = 3 and scale parameter *λ* = 2 was chosen. Moreover, the values *N* = 4, 6, 8, 10 were chosen for the approximating polynomials. In this case, as *N* increases the goodness-of-fit increases. However, note the fluctuations and negative values of the polynomial approximation for different choices of *N* on the displayed interval. This shows that the possible choices of interval [*a*′, *b*′], for which the polynomial approximation needs to be positive, depends on *N*.

However, note the fluctuations and negative values for the polynomial approximation for certain choices of [*a*′, *b*′] for each choice of *N*.

## Comparison with Gram–Charlier series approximations

A common type of polynomial-series expansion for PDFs is the Gram–Charlier type. In short, when the true PDF *f*(*x*) of a random variable *X* is unknown, it is approximated with a PDF of the form:

$$g\left(x\right)=p_{N}\left(x\right)\phi \left(x\right)$$ (11)

where *ϕ*(*x*) is the PDF for a Normal distribution with zero mean and unit variance. The polynomial *p*_*N*_(*x*) is then of the form:

$$p_{N}\left(x\right)=\sum_{i=1}^{N}c_{i}H_{i}\left(x\right)$$ (12)

where *H*_*i*_(*x*) represents Hermite polynomial of order *i*.

The expansions, in terms of the coefficients *c*_*i*_, can be presented in various equivalent ways, using cumulants or moments ([11], Chapter 6). Introducing *γ*_1_ and *γ*_2_, the skewness and (excess) kurtosis (with *μ*_2_, *μ*_3_ and *μ*_4_ being the second, third and fourth central moments, giving the relations $\gamma_{1}=\mu_{3}/\mu_{2}^{3/2}$ and $\gamma_{2}=\mu_{4}/\mu_{2}^{2}-3$.) of the distribution, we can write in a compact way, when *X* is a standardized random variable (zero mean and unit variance):

$$p_{4}\left(x\right)=1+\frac{\gamma_{1}}{6}H_{3}\left(x\right)+\frac{\gamma_{2}}{24}H_{4}\left(x\right).$$ (13)

This is referred to as Gram–Charlier type A. The related Edgeworth expansion involves one more Hermite polynomial, while keeping the number of parameters constant:

$$p_{6}\left(x\right)=1+\frac{\gamma_{1}}{6}H_{3}\left(x\right)+\frac{\gamma_{2}}{24}H_{4}\left(x\right)+\frac{\gamma_{1}^{2}}{72}H_{6}\left(x\right).$$ (14)

In the sequel, we use the former type, Eq (13), as will be commented upon shortly. Examples of successful modelling are found in various applications, see for instance [25, 26].

These expansions have a serious drawback, also apparent for the polynomial distribution: the approximation could result in negative values. Conditions on ensuring positive outcome were given by Barton and Dennis [27], where it was shown that for the Edgeworth expansion, Eq (14), the range for *γ*_1_ and *γ*_2_ over which positivity of the approximation is guaranteed is smaller than for the Gram–Charlier one [28].

Moreover, from a practical point of view, the series must have a finite number of terms. Use of higher-order terms does not necessarily guarantee a better result [11, 29]. For instance, in [11] a numerical example is given where actually four- and five-term series are worse than the three-term; each giving a negative frequency at a high value and a second mode at a low value (contrary to the data).

**Fig 3:**
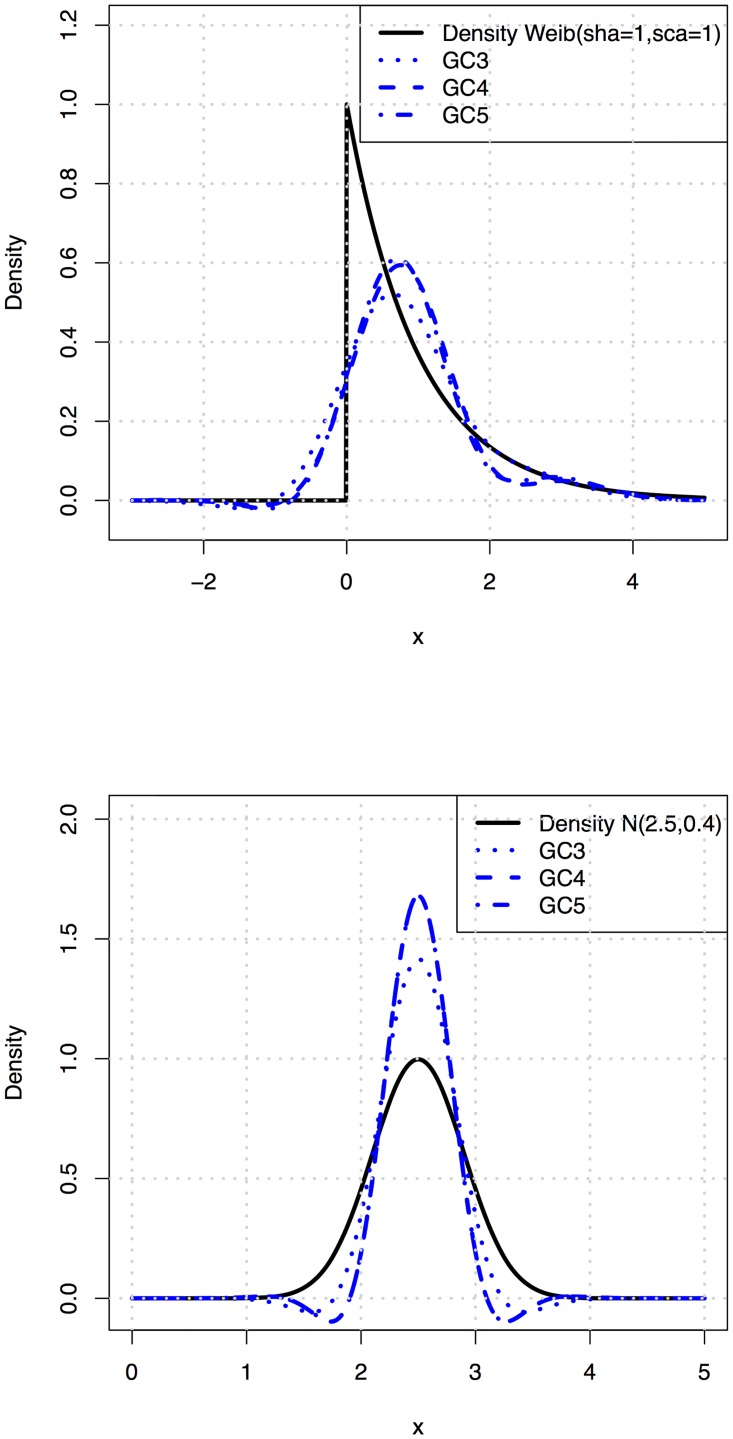
Examples of Gram-Charlier series expansions of a Weibull distribution (left) and a Normal distribution (right).

We consider cases with the Gram–Charlier expansions applied to some of the distributions and parameter settings presented in Fig 3. Note that these examples do not represent a situation with zero mean and unit variance; a transformation (and back transformation) had to be done to get the right scaling.

In Fig 3, we compare the cases of a two-parameter Weibull distribution (shape parameter *k* = 1, scale parameter *λ* = 1) and a Normal distribution with mean 2.5 and standard deviation 0.4. For each distribution, expansions with 3, 4 or 5 Hermite terms were considered. For the Weibull case, we note that for this particular parameter combination, the behavior at origin is difficult to capture for the various expansions and we get contributions of probability also at negative values, in addition to negative values of the density function itself. Note that the polynomial approximation proposed in the present paper approximates the true density more closely, cf. Fig 1. For the Normal distribution, we note that the overall mode behavior is found, but there are negative values in the resultant density.

## Data application example

**Fig 4:**
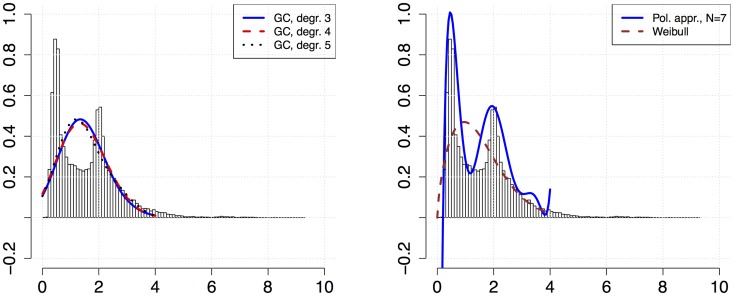
Histograms of household electricity use data with Gram–Charlier PDF (left), Weibull PDF (right) and polynomial PDF (right) fit. The polynomial distributions were computed from numerically calculated moments on the interval *a* = 100, *b* = 5000. A Weibull or Log-Normal PDF is usually a typical choice for this type of data [2].

As an example of an application of the procedure, we consider a data set on measured household electricity use with ten-minute resolution for a detached house over one year, see [2, 30]. In power systems modeling it is conventional to fit a unimodal distribution family such as a Log-Normal or Weibull distribution to this type of data, although the data set does not always correspond to an ideal unimodal distribution [2]. The data set is here fitted with Weibull, Gram–Charlier and polynomial distributions, and the resulting PDFs are shown in Fig 4. The moments for the data set were obtained using Eq (3). For the Gram–Charlier expansion, moments were estimated from data by the routine emm from the R package actuar. The parameters of the Weibull distribution were estimated with the maximum likelihood estimate routine wblfit in Matlab. For the computation *a* = 100 and *b* = 4000 were used, which were also assumed as upper and lower limits for the polynomial approximation, as to represent reasonable lowest and highest power use for the household.

Based on the calculations we may conclude that the results from the Gram–Charlier series approach is similar to the Weibull distribution by displaying unimodal characteristics, albeit with a different location of the peak. For higher number of terms in the Gram–Charlier series approach the resulting PDF becomes negative in the interval and displays a rugged behavior. The polynomial distribution, on the other hand, captures both peaks of this particular data set. However, it is negative for values near zero, but this is not an issue as the valid interval [*a*′, *b*′] of the polynomial distribution is chosen to be [100, 4000], on which it is positive. The polynomial distribution also has some extra fluctuations between 3 kW and 4 kW, which is similar to Runge’s phenomenon [31], and stems from the polynomial approximation characteristics of the approach, which is analogous to polynomial interpolation.

## Integro-differential equation application: The Smoluchowski coagulation equation

The procedure may be used to approximate solutions to certain differential and integro-differential equations whose solutions are probability density distributions. The formal criteria are that the solution is a probability density distribution and that *N* moments for the solution are available.

One example of this is the Smoluchowski coagulation equation (SCE) [32], for which moments of the solution can be obtained for certain cases, and thus the moment transform can be used to approximate the resulting probability distribution. The Smoluchowski coagulation equation is [33]:

$$\begin{array}{l} \begin{array}{l} \frac{\partial f}{\partial t}=\frac{1}{2}\int_{0}^{m}K\left(m-m^{\prime },m^{\prime }\right)f\left(m-m^{\prime },t\right)f\left(m^{\prime },t\right)\text{d}m^{\prime }-\\ \;-f\left(m,t\right)\int_{0}^{\infty }K\left(m,m^{\prime }\right)f\left(m^{\prime },t\right)\text{d}m^{\prime }, \end{array} \end{array}$$ (15)

which describes the process of a particle of mass *m* joining with another particle of mass *m*′, given an initial mass distribution *f*(*m*, 0) and a coagulation kernel *K*(*m*, *m*′). Here it is implicitly assumed that the kernel *K*(*m*, *m*′) is such that exact equations can be formulated for integer-order moments, although the equations may, in principle, include non-integer order moments.

With the moments *M*_*n*_ of *f*(*m*, *t*) as previously defined, the moment equations corresponding to the SCE are

$$\frac{dM_{n}}{dt}=\frac{1}{2}\int_{0}^{\infty }\int_{0}^{\infty }K\left(m,m^{\prime }\right)\;f\left(m,t\right)f\left(m^{\prime },t\right)\;\left[{\left(m+m^{\prime }\right)}^{n}-m^{n}-{\left(m^{\prime }\right)}^{n}\right]\;dm^{\prime }\;dm.$$ (16)

For kernels *K* which are expressible as a combination of powers of the particle masses *m* and *m*′, the right hand side will only contain moments of *f*(*m*, *t*). However, due to the term on the right-hand side containing the (*m* + *m*′)^*n*^ − *m*^*n*^ factor, it is only possible to construct equations for integer-order moments regardless of the kernel *K*.

The SCE has three known exact analytical solutions. These are for a constant (*K* = *K*_0_), linear (*K* ∝ *m* + *m*′) and product (*K* ∝ *mm*′) kernel [34]. With $K\left(m,m^{\prime }\right)=\frac{1}{2}\left(m+m^{\prime }\right)$, *M*_0_(0) = *M*_1_ = 1 and an initial configuration corresponding to infinitesimally small initial clusters (note that *f*(*m*, 0) has a singularity at *m* = 0), the solution of the SCE is [33, 34]:

$$f\left(m,t\right)=\frac{1}{\sqrt{2\pi }\;m^{3/2}}\exp\left(-\frac{m}{2}\;e^{-2t}-t\right).$$ (17)

Multiplying with *m*^*n*^ and integrating over *m* ∈ [0, *a*], an expression for the truncated moments ${\tilde{M}}_{n}$ is obtained,

$${\tilde{M}}_{n}\left(a,t\right)=\int_{0}^{a}m^{n}\;f\left(m,t\right)\;dm=\frac{2^{n-1}\;e^{2(n-1)\;t}}{\sqrt{\pi }}\left[\Gamma \left(n-\frac{1}{2}\right)-\Gamma \left(n-\frac{1}{2},\frac{a}{2}\;e^{-2t}\right)\right]$$ (18)

which provides the time evolution of any truncated moment of arbitrary order *n*. Using the above expression for ${\tilde{M}}_{n}$ a polynomial approximation of *f*(*m*, *t*) should be possible to obtain for a given time *t*. Since an exact solution exists for this particular case (see Eq (17)), it is possible to test the reliability of the method as a way of obtaining approximate solutions to the SCE.

Before the results of this example are presented, a known but not yet fully understood weakness of the procedure outlined in this paper, is mentioned. Recreating power-laws is problematic and usually leads to the occurrence of “wiggles” in the polynomial approximation. This is analogous to Runge’s phenomenon and probably related to the same [31]. However, if a power-law component can be identified, it can be removed before the transformation is made. In the case considered above, this would correspond to multiplication by *m*^3/2^, before the moments ${\tilde{M}}_{n}$ are calculated. Then, dividing the resultant polynomial with *m*^3/2^ will yield an approximation of *f*(*m*, *t*) which does not suffer from Runge’s phenomenon, except at the upper end of the mass scale where *f*(*m*, *t*) → 0.

**Fig 5:**
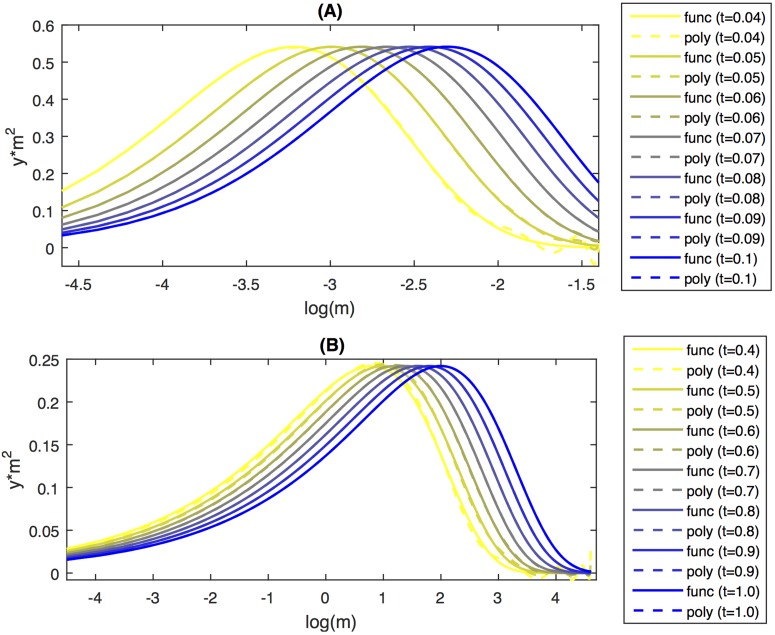
A plot of approximate solutions to the SCE for constant kernel (A) and for linear kernel (B). The approximation was based on the moment transform and polynomial degree *N* = 8.

The results for polynomial approximation of the solutions for constant (A) and linear kernel (B) is shown in Fig 5. By visual inspection, the polynomial approximation is reasonable fit to the analytically calculated results. For time 0.04 in the constant kernel case and time 0.4 in the linear kernel case there is some deviation, which is likely due to Runge’s phenomena. For other choices of mass-range and time steps the deviations could be dominant. This suggests that developing an internal check for convergence in the procedure would be helpful, in particular when there are no analytical solutions or even numerical simulations available to compare with.

## Practical issues

From computations with the polynomial distribution procedure it is possible to conclude two practical issues of particular interest:

1. Numerical computations of the inversion of **M** can become problematic as *N* increases.
2. When the PDF is close to zero there is a high tendency for mismatch.

Issue 1 arises since the Hankel matrix, and in particular the Hilbert matrix, are ill-conditioned [35]. This can be alleviated by using the algorithm for inverting Hankel matrices [21], or using the inversion formula for the Hilbert case of *a* = 0 and *b* = 1, when applicable [22]. The second issue has characteristics similar to Runge’s phenomenon, see [31] for more information on that. This issue appears not to be easily resolved, it is instead instructive to use the algorithmic step 9 and adapt *N* and the interval [*a*, *b*] for the moments, or the interval [*a*′, *b*′] for the distribution if admissible, so that the mismatch can be minimized.

## Discussion

We have suggested a simple procedure for estimating an *N*-order polynomial approximation to a known or unknown distribution based on *N* statistical moments of the distribution. The procedure offers a possibility to use polynomial distributions as simple approximations of distributions which alleviates the necessity for identifying and defining distribution families or even particular parameters of interest. This could be particularly useful for approximating non-unimodal distributions such as the aforementioned examples of a bimodal distribution and the distribution of household electricity use.

The procedure is based on the perhaps simplest possible polynomial approximation. For a potentially better fit it is possible—without changing the framework of the procedure—to adopt alternative polynomial expansions, such as a Chebychev polynomial or a Hermite polynomial, like in the Gram-Charlier series expansion. Alternatively, expansions and moments that are not centered at the origin could be used. It is suitable to let the goodness-of-fit and applicability determine the choice of polynomial expansion. However, with alternative polynomial expansions there is no guarantee that the matrix remains Hankel type, or even that it is invertible. Another possibility for improved goodness-of-fit is to use different order (e.g., non integer-orders) of moments, since there is no requirement within the framework of the procedure that the moment orders must be consecutive natural positive integers, even if integer-order moments are most commonly used in statistics.

Since the polynomial approximation only has satisfactory goodness-of-fit within a limited interval [*a*′, *b*′], the procedure is perhaps most suitable for applications which are defined on truncated probability distributions.

More advanced analysis of the convergence of the procedure is needed, in particular results which provide an explicit internal check for convergence.

Detailed investigations regarding the overall fitness of the procedure compared with other nonparametric probability density estimators such as kernel-density estimation and B-splines could be interesting to pursue for various types of distributions. For such investigations model complexity would be an interesting measure alongside approximation fitness, where perhaps the Akaike information criterion (AIC) could be a useful combined measure of this [17].

Generally, the applicability to differential or integro-differential equations is largely unknown, since only an example was given in this paper. More detailed investigations into this could prove fruitful. It is conjectured that this procedure can be generally useful for finding approximations to differential equation solutions when moments of the solution are available.

## Supporting information

S1 Dataset Data set on household electricity use used in this paper. (TXT)
