# Correction: Polynomial probability distribution estimation using the method of moments

**DOI:** 10.1371/journal.pone.0219530

**Published:** 2019-07-05

**Authors:** Joakim Munkhammar, Lars Mattsson, Jesper Rydén

In the Procedure section there is an error in equation six in the bottom row of the matrix **M**. Please view the complete, correct equation here:

$$M=\left[\begin{array}{cccc} b-a & \frac{b^{2}-a^{2}}{2} & \cdots & \frac{b^{N+1}-a^{N+1}}{N+1}\\ \frac{b^{2}-a^{2}}{2} & \frac{b^{3}-a^{3}}{3} & \cdots & \frac{b^{N+2}-a^{N+2}}{N+2}\\ \frac{b^{3}-a^{3}}{3} & \frac{b^{4}-a^{4}}{4} & \cdots & \frac{b^{N+3}-a^{N+3}}{N+3}\\ \vdots & \vdots & \ddots & \vdots \\ \frac{b^{N+1}-a^{N+1}}{N+1} & \frac{b^{N+2}-a^{N+2}}{N+2} & \cdots & \frac{b^{2N+1}-a^{2N+1}}{2N+1} \end{array}\right],w=\left[\begin{array}{c} w_{0}\\ w_{1}\\ w_{2}\\ \vdots \\ w_{N} \end{array}\right],E=\left[\begin{array}{c} E_{f}\left[X^{0}\right]\\ E_{f}\left[X^{1}\right]\\ E_{f}\left[X^{2}\right]\\ \vdots \\ E_{f}\left[X^{N}\right] \end{array}\right].$$
